# Scan of the postgraduate educational environment domains questionnaire: a reliable and valid tool for the evaluation of educational environment in postgraduate medical education

**DOI:** 10.1186/s12909-024-06125-3

**Published:** 2024-10-11

**Authors:** Sadrian Seyedhassan, Moosavi Mahsa, Ostovarfar Jeyran, Amini Mitra, Ghaderpanah Rezvan, Mokhtarpour Sedigheh

**Affiliations:** 1 Studens Research committee, School of Medicine, Shiraz University of Medical Sciences, Shiraz, Iran; 2 Clinical Education Research Center, Shiraz University of Medical Sciences, Shiraz, Iran; 3 MPH Department, Medical School, Shiraz University of Medical Sciences, Shiraz, Iran

**Keywords:** Reliability, Validity, Postgraduate Medical Education

## Abstract

**Background:**

The educational environment plays a critical role in shaping learners’ perceptions and experiences in medical education. Evaluating and enhancing the quality of this environment is essential for the continuous improvement of medical training programs. The Scan of the Postgraduate Educational Environment Domains (SPEED) is a concise instrument that assesses three domains of the educational environment. This study aimed to translate the SPEED questionnaire into Persian and evaluate its validity and reliability in the context of postgraduate.

**Methods:**

A cross-sectional study was conducted with 200 first and second-year medical residents. The Persian translation of the SPEED questionnaire was assessed for content validity, and confirmatory factor analysis was performed to evaluate its structural validity. Cronbach’s alpha coefficient was calculated to assess internal consistency reliability.

**Results:**

The Persian-translated SPEED questionnaire demonstrated satisfactory content validity, with all items exceeding the minimum acceptable values for content validity ratio and index. Confirmatory factor analysis indicated an acceptable fit for the 3-dimensional structure of the SPEED instrument. Internal consistency reliability analysis showed high reliability for the content, atmosphere, and organization domains.

**Conclusion:**

The Persian-translated version of the SPEED questionnaire is a valid and reliable tool for assessing the domains of the educational environment in postgraduate medical education.

## Introduction

Education serves as a fundamental pillar for the progress of society, requiring an ongoing process of evaluation, enhancement, and quality assessment to ensure alignment with the evolving needs and challenges of the educational system [1]. Within this context, the educational environment plays a crucial role in shaping learners’ perceptions of quality through its physical, emotional, and intellectual interactions with trainees [2]. While the medical educational environment shares certain similarities with other higher education settings, it also presents unique complexities and obstacles [3]. Therefore, further exploration and research are essential to thoroughly evaluate this environment.

The educational environment significantly impacts various aspects of clinical education programs, such as trainee satisfaction, attendance, motivation, well-being, and overall quality of life [4]. Recent studies have shown that trainee satisfaction with the training environment plays a crucial role in fostering professional engagement and cultivating positive attitudes towards the profession [5]. Additionally, trainee satisfaction serves as an important indicator for assessing the impact of faculty performance and programs on the overall training experience [5]. Evaluation is an essential component of higher education management, employing research and assessment methods to determine quality ratings, facilitate decision-making, collect relevant data, and establish benchmarks for program value, quality, and effectiveness [1]. A carefully defined and systematic evaluation process can identify strengths, address weaknesses, and provide a foundation for informed decision-making and educational planning. Ultimately, this process can contribute to the improvement of education standards in higher education institutions [6].

Multiple evaluation tools are available to assess the medical education environment and its impact on the learning process. Existing instruments, such as the Dutch Residents Educational Climate Test (D-RECT) [7], the Canadian Medical Education Directions for Specialists (Can-MEDS) [8], the Postgraduate Hospital Educational Environment Measure (PHEEM) [9], and the Dundee Ready Education Environment Measure DREEM [10], are relatively extensive, consisting of 40–50 questions or statements. Participants often do not have enough time or patience to answer the questions.

To address this limitation, Schönrock-Adema et al. (2015) developed a robust theoretical framework that focuses on assessing three critical domains of the educational environment using the tool “The Scan of the Postgraduate Educational Environment Domains” (SPEED), which comprises only 15 questions [11, 12]. Other strengths of this questionnaire include its comprehensive and innovative development based on a correct theoretical framework and previous research, involvement of stakeholders, and adherence to expert recommendations in this field [12].

In this study, we used the SPEED because it is specifically designed for postgraduate education, concise, and based on Moos’ theory of human environments, making it particularly suitable for the medical education context [12, 13].

The validity of the SPEED instrument was tested by Schönrock-Adema et al. (2015) through a web-based survey administered to residents in general hospital and a college hospital in the Netherlands. However, the authors recommended that future studies further investigate the instrument’s validity [12]. In response to this suggestion, Malau-Aduli et al. (2019) assessed the validity of the SPEED questionnaire in a postgraduate training setting in rural general practice [5]. While SPEED is considered comprehensive and reasonably concise, its validity and reliability have not been fully established. Further research is needed to validate the instrument and ensure its reliability in different medical education contexts.

### Aim of the study

The objective of this study is to translate the SPEED questionnaire into Persian and evaluate its validity and reliability within the context of postgraduate medical education in Iran. By undertaking this endeavor, the study will contribute to the expanding body of research on the assessment of the medical educational environment and provide a valuable tool for evaluating and improving the quality of postgraduate medical education at Shiraz University of Medical Sciences.

## Method

### Sample size calculation and participant recruitment

This cross-sectional study was conducted in 2021 to investigate the reliability and validity of the Persian version of the SPEED questionnaire among a sample of 200 first and second-year medical assistants in government and teaching hospitals of Shiraz University of Medical Sciences. Students were selected from the assistants of clinical fields such as radiology, family medicine, social medicine, ophthalmology, psychiatry, etc. The sample size for this study was determined using the N/P ratio, which states that the ratio of items to participants should be at least 1/10. This means that there should be a minimum of 10 respondents for each item in the questionnaire [14]. To account for potential attrition, a 20% increase was applied, resulting in a final sample size of 200 participants [15]. We obtained permission from the main producers of the questionnaire to use it and conduct validity and reliability in Iran. Ethical clearance was obtained from the Shiraz University of Medical Sciences before starting the study. Then, two of the researchers went to the government and university-affiliated hospitals and distributed the questionnaires among the medical assistants. It was explained to them that their participation in the study was completely voluntary. Informed consent was obtained from all the participants, and measures were taken to ensure confidentiality and anonymity.

### Data gathering tool and procedure

In this study, the Persian version of the SPEED questionnaire was used in the first section to respond to the study’s purpose. The SPEED questionnaire, as designed by Schönrock-Adema J et al., contains a total of 15 items with a 4-point rating scale (ranging from 0 to 3) and is appropriately brief. It assesses three domains: 1) Content: Themes related to appraisal and feedback, monitoring of progress, teaching style, independence and responsibility, and purposefulness (questions 1–5); 2) Atmosphere: Themes of accessibility, team spirit, relationships and atmosphere, support, and respect (questions 6–10); and 3) Organization: Themes of learning goal-oriented organization, organization of supervision, and clarity of tasks (questions 11–15) [12]. The DREEM (Dundee Ready Education Environment Measure) questionnaire consists of 50 items and is designed to assess undergraduate students’ perceptions of the educational environment [16]. On the other hand, the PHEEM (Postgraduate Hospital Educational Environment Measure) questionnaire, comprised of 40 questions, is used to evaluate the perceptions of hospital-based residents. This instrument measures the overall quality of the learning environment across three subscales: “role autonomy,” “quality of teaching,” and “social support” [17]. The new instrument discussed in this paper represents a merger of a solid theoretical framework and aspects of existing instruments to evaluate the quality of the postgraduate medical education (PGME) environment.

### Persian translation of the SPEED

The translation process for the SPEED instrument adhered to the four sequential translation and back-translation stages recommended by Chen et al. [18]. Emphasis was placed on ensuring conceptual accuracy rather than literal translation, employing a linguistic approach that was readily acceptable to Persian-speaking participants. Two independent translators, both native Persian speakers fluent in English, were involved in translating the questionnaire into Persian. One translator was familiar with SPEED, while the other had no prior knowledge of the questionnaire’s subject matter. Disputed points in the translation were resolved through discussion between the two translators. To ensure the questionnaire’s validity, the Persian version was back-translated into English by two different translators, both native English speakers. One translator was familiar with SPEED, while the other was not. The entire translation and back-translation process, along with coordination between translators, was supervised and documented by the authors.

### Statistical analysis

To establish the face validity of the Persian version of the SPEED questionnaire, the questions were assessed for their writing style, clarity, and fluency. Content Validity Index (CVI) and Content Validity Ratio (CVR) were utilized to confirm the accuracy and conceptual alignment between the original and translated SPEED questionnaires, ensuring equivalence with the original English version. For content validity determination, 15 experts were invited to rate each questionnaire item based on Lawshe’s method, assessing the necessity of the questions [19]. The questionnaires were distributed to faculty members who provided their opinion on the necessity and appropriateness of each question using a Likert scale (necessary, useful but not necessary, or not necessary).

All statistical analyses were performed using SPSS version 23 software (IBM Corp., Armonk, NY, USA) and LISREL version 8.8 (Linear Structural Relations) software. The significance level for the tests was set at 0.05 or less. Cronbach’s alpha coefficient was calculated to determine the internal consistency reliability of each subscale and the overall questionnaire. A value of 0.70 or higher indicated acceptable reliability for each subscale [20]. Confirmatory Factor Analysis (CFA) was conducted to examine the validity of the factor structure. Several criteria, such as Chi-square statistics, Root Mean Square Error of Approximation (RMSEA), Comparative Fit Index (CFI), Incremental Fit Index (IFI), Goodness of Fit Index (GFI), Normed Fit Index (NFI), and Adjusted Goodness of Fit Index (AGFI), were employed to evaluate the model’s goodness of fit.

## Results

A total of 200 questionnaires completed by first and second-year residents were analyzed. The findings indicated that the original version of the SPEED instrument could be effectively applied to the Persian translation of the scale.

### Construct validity

The content validity was quantitatively assessed using two coefficients: the Content Validity Ratio (CVR) and the Content Validity Index (CVI). The CVI values for all questionnaire items exceeded 0.79, indicating robust content validity. Additionally, the CVR, as determined by experts, was greater than 0.62 for all questions. Therefore, the questionnaire demonstrated strong content validity, with all items having CVI values of 0.79 or higher, and the overall scale having a CVR of 0.62. (see Table 1)

**Table 1:** Content validity

Items		CVI	CVR
Content	The feedback provided by my supervisor is focused on my strengths and weaknesses	0.83	0.63
	In this rotation, evaluations are useful discussions about my performance	0.81	0.86
	My supervisors are all in their own way positive role models	0.86	0.86
	The level of autonomy given to me is appropriate to my level of training	1	1
	The training in this post prepares me for my future career	1	1
Atmosphere	The supervisors are approachable and helpful	0.86	1
	Supervisors, nursing staff, other allied health professionals and residents work together as a team here	1	0.73
	There is (are) NO attending physician(s) who have a negative impact on the educational climate	1	0.82
	My supervisor supports me in difficult situations (e.g.handover)	1	0.81
	The supervisors are respectful towards residents	0.80	1
Organization	Teaching and learning are emphasized in this department	0.79	1
	Good clinical supervision is available at all times	0.79	0.79
	The staff is clear about my duties and responsibilities	1	1
	My program director reserves time to supervise/counsel me	1	0.89
	My program director prevents me from having to perform too many tasks irrelevant to my learning	1	1

To assess the goodness of fit of the final model of the SPEED tool, confirmatory factor analysis (CFA) was conducted. The results presented in Table 2 indicate that the Chi-square Ratio of the Degrees of Freedom (CMIN/DF) is less than 3, suggesting a good fit for the model. Additionally, the Goodness-of-Fit Index (GFI), Adjusted Goodness-of-Fit Index (AGFI), Normative Fit Index (NFI), Comparative Fit Index (CFI), and Incremental Fit Index (IFI) all exceeded 0.9, meeting the fit standards. The RMSEA index was calculated as 0.082, which falls within the range of 0.08 to 0.10, indicating a moderate fit [21].

We used the Root Mean Squared Error (RMSE) to measure the standard deviation of the prediction errors. Comparative Fit Index (CFI) values above 0.90 indicate a good fit. Tucker-Lewis Index (TLI) values above 0.90 indicate a good fit. Root Mean Square Error of Approximation (RMSEA) values below 0.09 indicate a reasonable fit.

**Table 2:** The indicators of fitness of the factor analysis of the SPEED tool

Structure	RMSEA	CMIN/DF	IFI	RFI	NFI	GFI	AGFI	CFI
Four-dimensional structure	0.082	2.63	0.93	0.90	0.91	0.92	0.90	0.91
Significance level	< 0.1	< 3	≥ 0.90	≥ 0.90	≥ 0.90	≥ 0.90	≥ 0.90	≥ 0.90

**Fig. 1:**
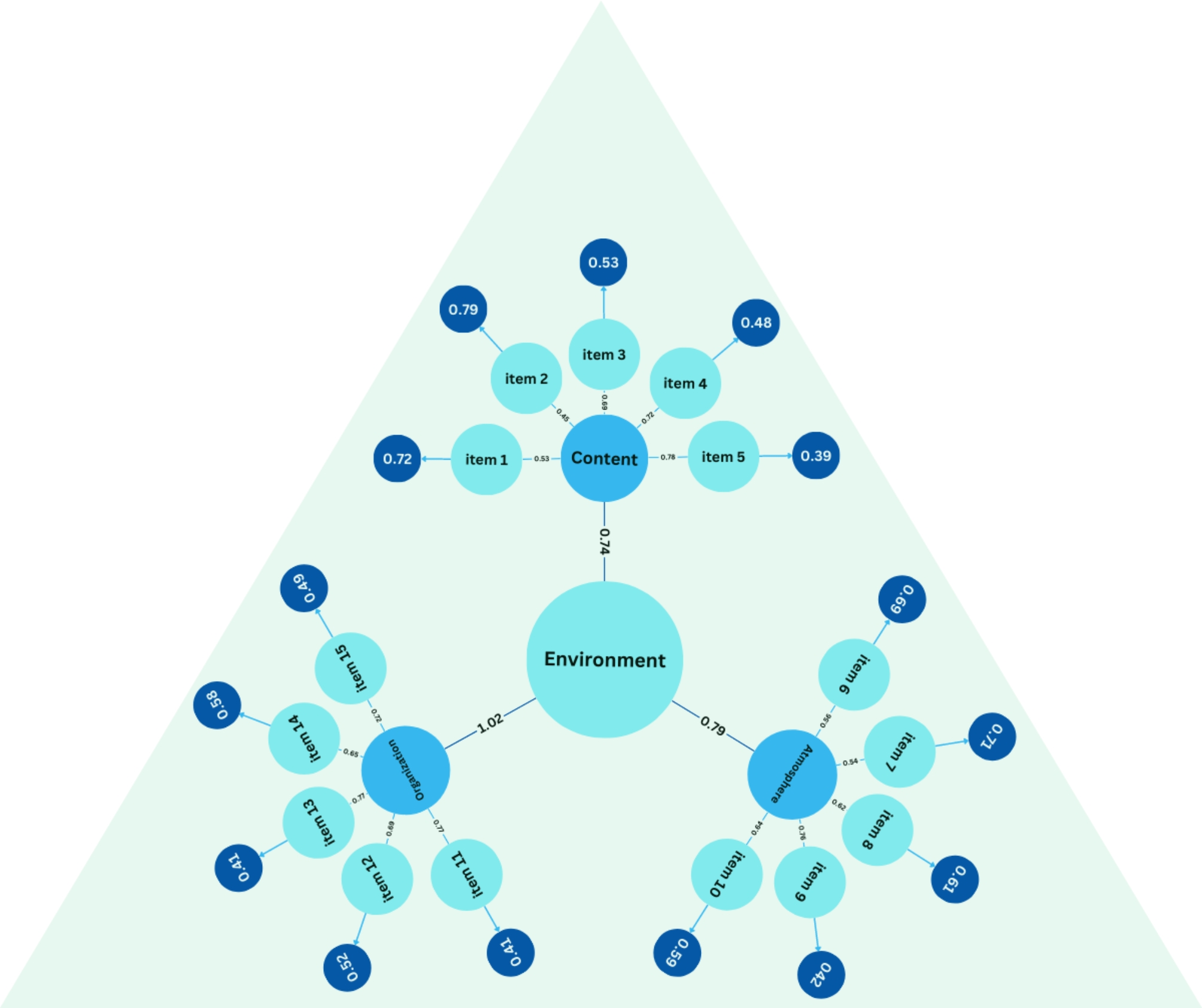
Factor structure of the SPEED tool. chi-square 229.04, dF 87, p-value 0.052, RMSEA 0.082

Figure 1 visually represents the factor structure of the SPEED instrument used in this study, demonstrating that all items exhibited moderate to high factor loadings (*p* < 0.001). This further supports the instrument’s validity. The diagram includes latent variables (factors), observed variables, and their relationships.

### Reliability

Cronbach’s alpha coefficient was used to assess the reliability of the SPEED questionnaire, and the results are displayed in Table 3. The obtained values indicate that the SPEED questionnaire exhibits high reliability, further affirming its consistency and stability.

**Table 3:** The reliability coefficients of the SPEED questionnaire

Domains	Item Numbers	Cronbach’s α
Content	1–5	0.768
Atmosphere	6–10	0.777
Organization	10–15	0.840
SPEED questionnaire	15	0.890

## Discussion

The primary objective of this study was to evaluate the validity and reliability of the Persian-translated version of the Scan of the Postgraduate Educational Environment Domains (SPEED) questionnaire. In particular, we aimed to determine whether the 3-factor model of the original SPEED questionnaire could be applied to the Persian translation of the scale. Our findings corroborate previous research, which indicated that a singular environmental component accounts for the variance observed in the instrument’s items [22]. This observation reinforces the complexity of the educational environment, suggesting that it is influenced by multiple, interrelated factors [23], consistent with the conclusions drawn by Roff et al. [16].

Interestingly, our results align with findings from other studies that demonstrated a correlation between most items in SPEED and the quality of care delivered in educational settings [24]. Consequently, it is not surprising that 15 items were consolidated into a single overarching factor. The original developers of the SPEED questionnaire posited that instrument performance could differ significantly due to the exclusion of specific items from their original 41-item version in our adapted 15-item scale [5]. This highlights the importance of item selection in constructing a reliable and valid questionnaire.

To further evaluate the reliability of the Persian-translated SPEED questionnaire, we focused on its internal consistency. Internal consistency measures the degree to which items within an instrument gauge the same concept or construct. It reflects the degree of interrelatedness among items within the test [15]. Following established guidelines, a Cronbach’s alpha coefficient exceeding 0.7 is indicative of acceptable reliability [25]. In our analysis, the reliability coefficients for the various SPEED domains ranged from 0.77 to 0.89, suggesting good internal consistency. These values not only affirm the reliability of the Persian version but also surpass the findings reported by Westein et al. [26] Additionally, they closely match the results from Malau-Aduli et al. [5] and Soemantri et al. [27]. In contrast, the study by Dorota Wojcik et al. reported Cronbach’s alpha values below 0.7 for one of the dimensions of the DREEM questionnaire [28] ), underscoring the robustness of the SPEED instrument in our context.

Additionally, we employed the Content Validity Ratio (CVR) to assess the extent to which experts agreed on the questions of the Persian-translated SPEED questionnaire. A CVR and Content Validity Index (CVI) value of 0.62 indicated good content validity [29]. In the Persian SPEED, the CVR value was 0.62 for the full scale, and the CVI values were at or above 0.79 for all items. The positive nature of these CVR and CVI values suggests that the Persian version of the SPEED questionnaire adheres to a logical and reasonable structure, consistent with the findings of Almanasreh et al. [29], thereby reinforcing our pursuit of a reliable educational assessment tool.

The suitability indicators derived from the confirmatory factor analysis model, as presented in Table 3, revealed that the components of the Persian version of the SPEED questionnaire have desirable conditions. To assess the fit of our model, we utilized various absolute fit indices, including CMIN/DF, GFI, and RMSEA, alongside comparative fit indices such as NFI and CFI. Noteworthy are our results, which yielded affable conclusions (CMIN/DF ≤ 3, CFI and IFI > 0.9, NFI value > 0.9, and RMSEA < 0.1), indicative of an acceptable model fit [21]. However, it is clear that further studies are essential to adapt the 3-subscale model of the SPEED questionnaire specifically for the medical students at Shiraz University. A thorough examination of the correlations between the loading estimates and the subscales in the path diagram substantiates that our data effectively aligns with the 3-factor model, echoing the findings of Schermelleh-Engel et al. [21].

Despite our study establishing a moderate effectiveness of the Persian-translated version of SPEED, we acknowledge that several limitations may influence the final assessment of these findings. Firstly, the research was conducted at a single institution, thereby restricting the generalizability of our conclusions. Moreover, the evaluation of the educational environment through three distinct dimensions in SPEED is a relatively new methodological approach. This necessitates that further studies be undertaken within Iran, as well as in additional international contexts, to enhance the understanding and comparison of the validity and reliability of our findings [30]. It is also pertinent to recognize that our study’s findings may not be applicable across different languages or educational environments, warranting cautious interpretation of the results. In pursuit of a more comprehensive understanding of our findings, further research is essential to ascertain the importance of replication and follow-up studies [31]. Finally, the responsiveness of the tool—essentially its capacity to reflect ongoing changes in the educational environment over time—was not evaluated in the study conducted by Schönrock-Adema J et al., and the scope of our present research did not encompass this critical aspect, necessitating future exploration. By addressing these areas, we can aspire to enrich the educational landscape and foster better learning environments across diverse contexts.

## Conclusion

The Persian-translated version of the SPEED questionnaire is a valid and reliable tool for assessing the domains of postgraduate educational environments. The implementation of this instrument is easy because it contains short statements in only 15 items. We recommend conducting this study on medical students at other medical universities in the country to further validate the instrument. The highlights of our work include the successful adaptation of the SPEED questionnaire to the Persian language and the demonstration of its validity and reliability in assessing postgraduate educational environments.

## Data Availability

The datasets used and analyzed during the current study are available from the corresponding author on request. The data are not publicly available due to privacy or ethical restrictions.
